# Are Patent Medicine Vendors Effective Agents in Malaria Control? Using Lot Quality Assurance Sampling to Assess Quality of Practice in Jigawa, Nigeria

**DOI:** 10.1371/journal.pone.0044775

**Published:** 2012-09-12

**Authors:** Sima Berendes, Olusegun Adeyemi, Edward Adekola Oladele, Olusola Bukola Oresanya, Festus Okoh, Joseph J. Valadez

**Affiliations:** 1 Liverpool School of Tropical Medicine, Liverpool, United Kingdom; 2 LAUTECH University Teaching Hospital, Osogbo, Osun State, Nigeria; 3 National Malaria Control Program Federal Ministry of Health, Abuja, Nigeria; Tulane University School of Public Health and Tropical Medicine, United States of America

## Abstract

**Background:**

Patent medicine vendors (PMV) provide antimalarial treatment and care throughout Sub-Saharan Africa, and can play an important role in the fight against malaria. Their close-to-client infrastructure could enable lifesaving artemisinin-based combination therapy (ACT) to reach patients in time. However, systematic assessments of drug sellers’ performance quality are crucial if their role is to be managed within the health system. Lot quality assurance sampling (LQAS) could be an efficient method to monitor and evaluate PMV practice, but has so far never been used for this purpose.

**Methods:**

In support of the Nigeria Malaria Booster Program we assessed PMV practices in three Senatorial Districts (SDs) of Jigawa, Nigeria. A two-stage LQAS assessed whether at least 80% of PMV stores in SDs used national treatment guidelines. Acceptable sampling errors were set in consultation with government officials (alpha and beta <0.10). The hypergeometric formula determined sample sizes and cut-off values for SDs. A structured assessment tool identified high and low performing SDs for quality of care indicators.

**Findings:**

Drug vendors performed poorly in all SDs of Jigawa for all indicators. For example, all SDs failed for stocking and selling first-line antimalarials. PMV sold no longer recommended antimalarials, such as Chloroquine, Sulfadoxine-Pyrimethamine and oral Artesunate monotherapy. Most PMV were ignorant of and lacked training about new treatment guidelines that had endorsed ACTs as first-line treatment for uncomplicated malaria.

**Conclusion:**

There is urgent need to regularly monitor and improve the availability and quality of malaria treatment provided by medicine sellers in Nigeria; the irrational use of antimalarials in the ACT era revealed in this study bears a high risk of economic loss, death and development of drug resistance. LQAS has been shown to be a suitable method for monitoring malaria-related indicators among PMV, and should be applied in Nigeria and elsewhere to improve service delivery.

## Introduction

Patent medicine vendors (PMVs) provide antimalarial treatment and care throughout Sub-Saharan Africa. They potentially play a critical role in the fight against malaria. However, systematic assessment of their performance quality is crucial for their management within the health system. Such an assessment can complement quality assurance through health facility assessments (HFAs) [1], [2], [3], [4], [5].

Malaria remains a leading cause of morbidity and mortality in Africa with an estimated 176 million cases and 709,000 deaths recorded in 2009. About 25% occur in Nigeria [6]. Although early diagnosis and prompt treatment is a principal strategy for global malaria control [6] only 26% of malaria cases in <5-year-old children in Nigeria were treated on the same or next day with “any antimalarial” and 3% with artemisinin-based combination therapy (ACT) [7]. In 2001 the World Health Organization (WHO) recommended ACT as the first-line treatment for uncomplicated malaria as a response to increasing Chloroquine (CQ) and Sulfadoxine-Pyrimethamine (SP) drug resistance [8]. ACT has meanwhile been officially adopted by most African countries including Nigeria (since 2005), but uptake remains slow [6].

Many people do not attend health facilities and choose self-medication through the often unregulated private retail market [7], [9], [10]. The WHO has encouraged participation of formal and informal private providers, such as PMVs, to implement government strategies for malaria control [3]. PMVs do not have formal pharmacy training but “sell orthodox pharmaceutical products on a retail basis for profit” [11]. Medicine vendors are ubiquitous in Africa and can be found even in remote villages and shantytowns [11], [12]. Because they are culturally and economically accessible, they are often the first choice for malaria treatment, especially in rural areas [2], [13], [14]. In rural Nigeria about 60% of mothers prefer to consult PMVs rather than other providers for treatment of childhood malaria [2]. Since PMVs form an established network, they provide an entry point for government interventions to distribute ACTs [15], [16]. ACTs have been declassified from prescription only to an over-the counter (OTC) medicine since 2005, thus allowing its dissemination by PMVS, whose patent and proprietary medicine vendor license only authorizes the selling of OCT drugs [17]. However, inadequate knowledge among PMVs and other structural or behavioral factors could lead to misdiagnosis, over- and under-treatment with drugs, and delayed referrals with increased risk of disease progression, toxicity and development of drug resistance [5], [12], [13], [18]. Drug resistance is a particular concern following introduction of ACTs because continued use of artemisinin-derivates as oral monotherapy could threaten the therapeutic life of ACTs by fostering artemisinin resistance [19], [20].

Malaria control strategies can take advantage of well-established PMVs’ close-to-client provision of care only if they address their technical failures and other factors that threaten the quality of their services [11]. Such action requires not only baseline assessments of PMVs’ performance to inform the design of interventions, but also regular monitoring and evaluation (M&E) of their services.

Several quantitative and qualitative studies have assessed the quality of antimalarial treatment and care among medicine sellers in Africa [1], [5], [9], [12], [13], [21], [22]. Nearly all were conducted prior to the adoption of ACT as the first-line regimen. Despite the lack of peer-reviewed publications about the availability and use of ACT by PMVs in Nigeria, limited evidence suggests that PMVs’ knowledge of the 2005 policy change endorsing ACTs as the new first-line antimalarial is low and ACT uptake is slow [4], [23].

To improve service delivery, research is needed to assess the adherence to international and national treatment guidelines by all health system stakeholders. Given the prevailing financial constraints, this would only be feasible if an evidence based method could be used that is not too costly and time-consuming. Here, we propose Lot Quality Assurance Sampling (LQAS) as such a method, because it uses a rapid, and inexpensive, but nevertheless precise technique that requires only small sample sizes to make meaningful statistical inferences. Lot Quality Assurance Sampling (LQAS) has been used successfully to assess the quality of care in other health arenas [24], [25], [26]. This paper uses LQAS for the first time to assess the quality of local PMV services for malaria treatment of the sick child. This work is one component of a larger study by the Nigeria Malaria Control Program (NMCP) [27] that assessed the quality of malaria and child health services. Here, we report on the availability and quality of malaria drugs and treatment advice among PMVs while demonstrating the use of monitoring technique that can be used recurrently for management purposes.

## Methods

### Sampling Design

States in Nigeria are divided into three Senatorial Districts (SDs); each includes about one-third of all health facilities (HFs). The study, implemented in Jigawa state, uses a two-stage LQAS sampling-design. Firstly, a random sample of all public and formal private primary and secondary HFs was selected in each SD. Secondly, one PMV located closest to the sampled HF was selected for study. If two or more PMV stores were of similar distance from the HF we chose one of them randomly. As patients leaving HFs with a diagnosis of malaria often have to purchase antimalarials at PMVs due to their non-availability in HFs [28], the greatest demand for antimalarials could be at the closest PMV. Therefore, a sample of PMVs located close to HFs could measure the quality of services in PMVs that experience the highest demand for antimalarials. While a two-stage design has been used in HFA-LQAS assessments [25], [26], [29], this PMV-LQAS design is used for the first time (Table 1).

**Table 1:** Jigawa State with three Senatorial Districts by total number of Health Facilities (HFs), PMVs, sample sizes, and alpha and beta errors for an LQAS decision rule of “d” = 5.

Senatorial District	Total Number of HFs (N)	Sample size of HFs (n)	Corresponding sample size for PMVs (n)	Alpha Error	Beta Error
Central	10	7	7	<0.001	0.083
North East	9	7	7	0.042	0.051
North West	9	7	7	0.042	0.051

### Using LQAS to Classify PMVs in Each SD

LQAS is a classification method originally developed for industrial quality control during the 1920s [30]. The principle is that a line supervisor examines a small random sample from a lot of recently manufactured goods, e.g. light bulbs on an assembly line. If the supervisor finds that the number of defective goods in the small sample exceeds a predetermined number, the whole lot is rejected. Otherwise it is accepted. The predetermined allowable number of defective goods is called “decision rule”. It is determined statistically to ensure that there is a high probability of correct classification, namely, that rejected lots contain a relatively high proportion of defective goods, and that accepted lots contain no or only a relatively low proportion of defective goods. However, there always remains a small risk of misclassification, as reflected in the level of statistical error. The “alpha error” is the probability of mistakenly rejecting a lot with no or only very few defective goods, and the “beta error” is the probability of mistakenly accepting a lot with too many defective goods.

LQAS was adapted to health sciences in the mid-1980s [29] and has meanwhile been widely used to monitor public health applications [31]. In our application, the lots are defined as the different SDs in Jigawa, including Central, North-East, and North-West-SD that manage all the health facilities within their geographical boundaries. The equivalent of a defective good would be a health facility with an unacceptable level of service quality. If after assessing a random sample “n” of all HFs in the SD, a certain predetermined number of HFs fail to show acceptable service quality, the SD is marked as an SD with “low” performance in need of special attention and resources for quality improvement.

Health systems managers of the different SDs in Jigawa were not only interested in the performance of HFs, but also in the performance of PMV stores reported in this paper. Therefore, for each SD a corresponding sample “n” of PMV stores was assessed. While in industrial quality control it might be easy to assess whether a good is defective, it is more complex to assess the service quality and performance of PMV stores. We used the PMV-LQAS tool adapted from previously validated tools [32] to assess different aspects of PMV performance, including the quality of PMV pediatric malaria treatment, the availability of antimalarials in their stores, and their training and knowledge about malaria prevention and treatment [33].

**Figure 1:**
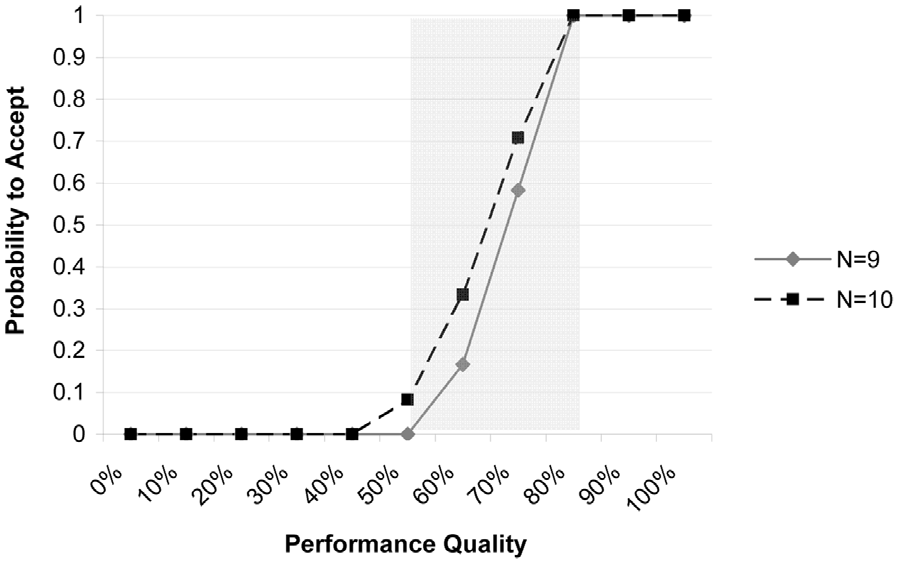
Operating Characteristic Curve for health facility assessments in Jigawa, Nigeria. Operating Characteristic Curve for health facility assessments with sample size n = 7 and a decision rule of d = 5 for the upper cut-off P_U_ = 80% and the lower cut-off P_L_ = 50% for Senatorial Districts with a total of N = 9 and 10 health facilities. The area in between the upper and lower cut-off is the “grey area”. Changing d would shift the curve to the left or right; increasing the sample size would result in a sharper gradient of the curve and a smaller grey area. However, no matter how large the sample size there will always be a grey area and a certain level of misclassification error. We selected n and d to reduce the two forms of misclassification errors; it was ensured that the alpha error or the probability of mistakenly failing to accept an SD with acceptable performance (≥80%) did not exceed a value of 0.10, and that similarly the beta error or the probability of mistakenly accepting an SD with unacceptable performance (≤50%) did not exceed 0.10.

The decision rule or cut-off “d” is the allowable number of PMV stores out of the sample of “n” stores in an SD that have to show acceptable performance for an SD to be classified in the “high” category with respect to a quality indicator. The cut-off “d” depends on the sample size (n), the level of misclassification error (alpha and beta error) that managers are ready to accept, and the thresholds that determine the minimum proportion “p” of PMV stores that have to show acceptable service quality for an SD to be classified as of “high” or “low” performance. Two thresholds need to be determined: an upper threshold “p_U_” above which SD performance will be classified as acceptable or “high”, and a lower threshold “p_L_” below which it will be classified as unacceptable or “low”. Between these two thresholds is the area commonly referred to as the “grey area” with SDs having intermediate performance. As LQAS makes only binary decisions, SDs with intermediate performance are also classified as high or low depending on their proximity to either threshold. All of the corresponding classification errors are depicted in an Operating Characteristic Curve (Figure 1). LQAS’s strength is in identifying SDs situated at the ends of the distribution of quality; it is therefore sensitive to identifying SDs which are among the worst of the worst with very low amounts of error and that can be targeted for improvement. The National Malaria Control Program (NMCP) set the upper threshold (p_U_) identifying acceptable performing SDs as 80% of PMVs using the national guidelines. This threshold level was decided by consensus during a stakeholder forum, in which the directors and M&E officers of the NMCP, as well as representatives of the World Health Organization and World Bank participated. It is informed by coverage information available in previous studies and is in line with the targets of the Nigerian 5-year Strategic Plan 2006–2010 that stated that an 80% coverage was both feasible and required to bring malaria under control [17]. The lower threshold (p_L_), was set 30% lower (which is typical), i.e. at 50%. The sample size (n) and decision rule (d) were selected to minimize the two forms of misclassification errors, so that the overall statistical error (α+β) was no more than 0.20.

For our PMV-LQAS design we used the hypergeometric formula to calculate the sample size of PMVs due to the small number of HFs in SDs [29]. This procedure generated a sample size of n = 7 PMVs for North West-SD and North East-SD, and a decision rule of d = 5, with an alpha error of 0.042, and beta = 0.051 for p_U_ = 80%, p_L_ = 50%. In Central-SD the parameters were the same except that alpha≤0.001, and beta = 0.083 (Table 1). These sample sizes can also be generated by entering all the parameters into a web-based LQAS sampling plan calculator using the hypergeometric distribution [34]. See [29] for more detail about LQAS.

### Data Collection and Ethics Statement

Data collectors were experienced Jigawa state health workers trained during 16–19 March 2009. They collected data during 20–26 March 2009 under the supervision of a state manager and an author, after receiving written informed consent from all participants. The study was approved by the Liverpool School of Tropical Medicine Research Ethics Committee, and by the Ministry of Health in Nigeria.

### Analysis

LQAS classified the three Jigawa SDs as having acceptable (p_U_ = 80%) or unacceptable (p_L_ = 50%) performance using a statistically determined decision rule (d = 5). We then aggregated the resulting data across the three SDs to calculate a performance proportion for the entire state. For a given performance indicator, the proportion of PMVs providing adequate services for Jigawa was estimated by weighting results from each SD by the total number of HFs in each SD and calculating the mean for the state (Jigawa). Confidence intervals were calculated using a finite population correction [35]. Analyses used Excel® and the Statistical Package for Social Sciences® (SSPS Version 15).

## Results

Data collectors interviewed 21 PMVs (7 in each SD). PMVs in the sample had store fronts rather than kiosks or a mobile outlet type of service delivery (100%); vendors tended to have secondary school education (38.1%) or above (57.1%, 5% no response), had a median of 5 years experience (interquartile range (IQR)–2.0–16.0), and attended to a median of 10 malaria cases (IQR 7.0–18.8) the day preceding the survey. Only one PMV was female (4.8%).

### Inputs and Processes: Stocks of Antimalarial Drugs and ITNs, and PMV Training

All three SDs failed to have an adequate proportion of PMVs stocking first-line antimalarials (Table 2). PMVs tended to stock drugs no longer recommended for treatment of uncomplicated malaria, especially Artesunate monotherapy, SP and CQ (Table 3). Similarly, 42.35% of PMVs stocked ACT brands that were not Ministry of Health (MOH) approved (Table 4). No SD displayed adequate PMV training on relevant malaria-related topics (Table 2).

**Table 2:** Input, Process and Output indicators and LQAS judgment for three Senatorial Districts (C-Central, NE-North East, NW-North West) in Jigawa, Nigeria, 2009.

Level	Indicator	LQAS Judgment for each district (n = 7 and d = 5)*			Weighted State aggregate	CI†
		C	NE	NW		
Input	PMVs with at least one first line antimalarial (ACTs) in stock‡	3 = Low	4 = Low	4 = Low	52.04%	±10.79%
	PMVs with at least one ITN/LLIN in stock‡	0 = Low	0 = Low	1 = Low	4.59%	±4.24%
Process	PMVs received malaria-related training in the past 3 years§	1 = Low	1 = Low	1 = Low	14.29%	±7.63%
Output	PMVs have knowledge of Government recommendations on treatment of uncomplicated malaria††	1 = Low	0 = Low	1 = Low	9.69%	±6.34%
	PMVs correctly described dosage of ACTs (AL and AA) for a 2 year old child§§	1 = Low	3 = Low	1 = Low	23.47%	±8.73%
	PMVs recognized at least 3 danger signs in children needing referral	1 = Low	1 = Low	1 = Low	14.29%	±7.63%
	PMVs who sold a first line antimalarial (ACTs) as a treatment for fever/malaria**	2 = Low	2 = Low	2 = Low	28.57%	±9.85%

* Number of adequately performing PMVs in the sample of n = 7 PMVs per SD and classification of SD as high or low performance based on the decision rule (d = 5).

† 95% confidence intervals for state estimates of coverage with finite population correction.

‡ as observed on the day of the survey.

§ PMV reported having had malaria-related training on at least three of the following components: treatment with ACTs, promotion of ITNs, referral for danger signs, disuse of chloroquine and SP for treatment.

†† PMV mentioned at least four of the following components of government recommendations: Treatment with ACTs, disuse of chloroquine and SP for treatment, disuse of oral artemisinin-based monotherapy for treatment, antimalarials to be given in single treatment packs, SP to be provided at least twice during pregnancy, treatment sought for febrile cases within 24 hrs of onset of symptoms.

§§ Interviewers brought the most common available brand of both types of recommended ACTs, showed them to the PMV and then asked them the dosage for a 2-year-old child for each drug. PMVs were not restricted from looking up dosage information if they wanted to do so.

¶ Recognizable danger signs to be named included convulsions, coma, severe vomiting, inability to drink or breastfeed, severe sickness/inability to sit or stand, difficulty in breathing/fast breathing, persistent fever, and lack of improvement or worsening of symptoms after two days.

** PMV reported that client looking for treatment of fever/malaria purchased a first line antimalarial (recommended ACTs) rather than a non-recommended antimalarial.

AL = Arthemether-lumefantrine, AA = Artesunate-amodiaquine, ITN = Insecticide Treated Nets, LLIN = Long Lasting Insecticide treated Nets.

**Table 3:** Antimalarials in stock on survey day and sold on the day before the survey at PMV stores in Jigawa, Nigeria, 2009*.

Policy	Antimalarials	In stock†					Sold on previous day‡				
		No. of PMV in SD§			Weighted State Aggregate	CI**	No. of PMV in SD§			Weighted State Aggregate	CI**
		C	NE	NW			C	NE	NW		
Recommended (ACTs)	Artesunate-amodiaquine	2	1	3	28.57%	±9.54	1	1	2	18.88%	±8.38
	Arthemether-lumefantrine	0	3	3	27.55%	±8.49	0	2	0	9.18%	±5.48
	Artesunate-mefloquine	1	0	1	9.69%	±6.34	1	0	0	5.10%	±4.71
Not recommended (oral mono-therapy)	Artesunate monotherapy	4	3	5	57.14%	±10.51	2	2	6	46.94%	±9.22
	Dihydroartemisinin monotherapy	0	0	0	0%	±0	2	0	0	10.20%	±6.09
	Arthemeter monotherapy	0	0	0	0%	±0	1	0	0	5.10%	±4.71
	Artemisinin monotherapy	0	0	0	0%	±0	1	0	0	5.10%	±4.71
Not recommended (other antimalarials)	Chloroquine	1	4	5	46.43%	±9.39	2	4	5	51.53%	±10.15
	Sulphadoxime-Pyrimethamine	4	2	5	52.55%	±10.22	4	1	5	47.96%	±9.61
	Amodiaquine	0	2	0	9.18%	±5.48	0	2	0	9.18%	±5.48
	Mefloquine	0	1	0	4.59%	±4.24	0	0	0	0%	±0
	Other	1	1	3	23.47%	±8.73	0	4	2	27.55%	±8.12

* Multiple responses were allowed; no LQAS judgements made because the data are stratified by response options as well as by stocking and selling of drugs that are recommended and non-recommended for treatment of uncomplicated malaria in Nigeria.

† As observed on survey day.

‡ Purchased by clients with fever/malaria on day before survey as reported by PMV.

§ Number of PMVs in the sample of 7 PMVs per Senatorial District (SD).

** 95% confidence intervals for state estimates of coverage with finite population correction.

**Table 4:** Available ACT brands observed on the survey day in PMV stores in Jigawa, Nigeria*.

Policy	Brand (generic)	No. of PMV in SD†			Weighted State Aggregate	CI‡
		C	NE	NW		
MOH approved	Coartem (AL)	0	3	2	22.96%	±8.12
	Arsuamoon (AA)	1	1	1	14.29%	±7.63
	Malact (AA)	0	2	0	9.18%	±5.48
	Nexanate (AA)	0	0	0	0%	±0
Not MOH approved	Others	2	4	3	42.35%	±10.44

* Multiple responses were allowed; no LQAS judgments made, because list includes adequate and inadequate behavior, i.e. stocking and selling of brands that are MOH approved and non-approved for treatment of uncomplicated malaria in Nigeria.

† Number of PMVs in the sample of 7 PMVs per Sub-District (SD) who had brands in stock as observed on survey day.

‡ 95% confidence intervals for state estimates of coverage with finite population correction.

MOH = Ministry of Health, AL = Arthemether-lumefantrine, AA = Artesunate-amodiaquine.

### Output Indicators: PMV Knowledge and Reported Purchasing Behavior

No SD reached the 80% performance standard for any of four output indicators. PMV knowledge of the Government recommendations on the treatment of uncomplicated malaria was poor in all SDs with less than 10% of PMVs being sufficiently familiar with the new treatment policy (Table 2). SDs also exhibited inadequate PMV knowledge on the correct dosage of ACTs for a two-year old child and on recognizable danger signs necessitating hospital referral (Table 2).

Finally, no SD had an adequate proportion of PMVs selling appropriate antimalarials. Less than 30% of PMVs reported having sold first-line antimalarials to malaria patients the previous day (Table 2), and about half of PMVs sold no longer recommended Artesunate monotherapy, CQ and SP (Table 3).

## Discussion

This study shows that PMVs, who are a relevant source of malaria treatment, performed poorly for all input, process and output indicators. On the input level ACT availability in PMV stores was low (52.04%), despite being higher than availabilities reported in the grey literature in 2007 for other states (Oyo, Kaduna and Enugu), where on average 8.5% of PMV shops stocked ACTs [36]. A 2008 report found that 19% of drug stores in Nigeria stocked ACT [37]. In contrast, during 2009 researchers in Enugu documented that ACT could be found in 89.6% of medicine retailers; results were not reported for PMVs alone and are difficult to compare with our findings [28]. While the availability of ACT in our study was poor, the availability of non-recommended antimalarials (SP (52.6%), CQ (46.4%), Artenusate monotherapy (57.1%)) was high. This is a pattern also found in the above mentioned studies [36], [37].

These findings are disturbing given that changes to Nigeria’s malaria treatment policy adopted ACT as the first-line treatment in 2005. At that time the National Administration of Food and Drugs Agency (NAFDAC) in collaboration with NMCP also declassified ACT from prescription only to an over-the-counter medicine, thus allowing its dissemination by PMVs. Simultaneously they discontinued the registration of artemisinin monotherapy, to prevent development of drug resistance [17], [38]. The drug availability described above and results from HFAs [28], suggest that these policies were not sufficiently implemented within the first four years following their introduction.

One reason PMVs to not adhere to the new guidelines in Nigeria might be due to the fact that the approved drug list given to PMVs upon registration and licensing had been last revised in 2003, and did not yet reflect the policy changes. The list did not indicate ACT, but rather CQ and SP as approved drugs for malaria treatment, which might have confused PMVs [39]. Besides this deficiency, and a lack of training and provider knowledge, limited consumer demand and the higher cost of ACTs might be additional reasons for low compliance with the new guidelines.

On the process level our PMV-LQAS survey found that only 14.3% of PMVs had been trained on malaria-related topics in the past three years, although PMV training had been included in the 2005–2010 strategic plan [17]. Previous studies report results that are less specific and thus difficult to compare, but suggest that the amount of training varies in different regions [28], [36]. A likely explanation for the absence of PMV training is that the Pharmacists Council of Nigeria (PCN), the regulating body for PMVs, was non-existent in Jigawa until a few months before this research. There has been little delineation of roles among the PCN, local government and other regulatory agencies about which of them should be responsible for monitoring PMVs. Problems of duplication of roles and bottlenecks surrounding the registration, licensing and monitoring of PMVs in the state could also partly explain other results reported above.

Given our findings about inputs and processes, poor performance on the output level was not surprising. In all three SDs the majority of PMVs was ignorant of the government’s recommendations on malaria treatment. In a previous report, PMVs knowledge on the change of policy related to first-line antimalarials was low, but interestingly, it differed across the states and regions, and might be explained by the fact that in one region, drug companies rather than the government were the main source of information on drug regulation [4]. Another report documented that knowledge of the new first-line regimens was lowest in drug stores, but only slightly better in formal private outlets and better, but still insufficient, in public health facilities [37].

Poor PMV knowledge about the disuse of oral artemisinin monotherapy for malaria treatment has been of particular concern. In Nigeria and elsewhere many PMVs still mistakenly consider artemisinin monotherapy as an ACT [21], [40]. Both the use of artemisinin monotherapy and incomplete dosages of ACT harbor a significant risk for emergence of drug resistance [19], [20], and might have contributed to the declining ACT efficacy recently observed on the Cambodia-Thailand border [41]. It is alarming that only 23.5% of PMVs knew the correct ACT dosage for a 2-year old child. In addition to the risk of drug resistance, incorrect dosages might lead to increased adverse events or treatment failure with grave health-related and economic consequences. Similarly, PMVs’ failure to recognize danger signs in children (as observed in this study and a previous one [37]) and to refer possible cases of severe malaria to higher-level care, can lead to increased child morbidity and mortality.

Given the observed drug availability pattern and the lack of PMV knowledge discussed above, our findings were not unexpected that PMVs favored non-recommended antimalarials. These results were consistent with a previous report [37]. While recent data on PMVs’ ACT dispensing behavior in Africa are scarce, pre-ACT era studies revealed incorrect dosing and irrational prescribing practices, including the dispensing of counterfeit and poor quality drugs; some of these studies suggested PMVs were influenced by advertising and profit motives [1], [5], [12], [42], [43]. However, PMVs are influenced by both supply side and demand side factors [11], [13], [44], and by the way drug distribution and health care is organized within the state [44].

These factors need further study to better understand how they influence PMV performance in the ACT era. Future surveys could include demand side indicators, and assess the knowledge, expectations, and service satisfaction of clients through observations of provider-client interactions or client exit interviews. It should also learn whether a client’s choice of antimalarials relies on PMV advice, or prescriptions by other providers, or their own decisions. Studies from the pre-ACT era revealed that most PMVs simply sold drugs that a customer requested, without giving advice and without requesting a prescription [9], [11]. A limitation of our study is that we did not record the price of drugs and explore its effect on provider and consumer behavior. Previous studies found that providers prescribe and dispense types and dosages of drugs not according to need, but according to a patient’s ability to pay, resulting in over-prescription or under-dosage of drugs [5], [18]. The assessment of the price of antimalarials at PMV stores will be all the more important in the future, with the implementation of structural interventions to improve ACT supply. Nigeria is one of the pilot countries of the Affordable Medicines Facility-malaria (AMFm) project launched in 2010 that aims to increase ACT availability by subsidizing ACTs purchased by eligible 1^st^-line buyers [45]. The AMFm Task Force has planned periodic ACT price and availability monitoring surveys at different outlets, which will hopefully also examine whether price reductions on top of the supply chain will be passed on to consumers at PMV stores.

Further studies should also examine the quality of drugs, either indirectly by checking storage conditions, packaging, expiry dates and the presence of a NAFDAC registration number [36], or directly by chemical testing [42]. Finally, future studies should document the availability and use of rapid diagnostic tests (RDT) by PMVs, should RDT be introduced for sale and use by PMVs. This option is under consideration by the MOH provided PMVs receive appropriate training, followed by regular supervision and quality assurance [38], [46].

LQAS has several strengths giving it an advantage over other common survey methods, such as cluster surveys, which have been used widely to assess immunization coverage and other health outcomes [47]. LQAS has been shown to be cost-effective for different public health applications, and especially if carried out in a decentralized way by trained health workers as part of routine monitoring, it requires less time and resources than cluster surveys [48], [49], [50]. LQAS data collection does not seriously compete for health workers’ time for providing health services, because only very small sample sizes are required to make performance judgments about a catchment area. Besides, sampling procedures and analysis, originally developed for use by assembly line supervisors of yesteryear, are simple and do not require substantial training [48]. As mentioned above, LQAS not only provides results on whether a lot or SD showed acceptable or unacceptable performance, but also allows combining results to provide the same state- and national-level information that other multistage surveys provide. Another interesting quality of LQAS is that it could be integrated into multistage cluster sampling surveys, as recently demonstrated for the malaria indicator survey [51]. These survey types are not intended, as is LQAS, to reveal variation among different local sub-areas [48], [52]. In our study we did not find much variation between different supervision areas, because all SDs performed poorly for all key indicators. However, LQAS has shown from previous studies that it is an excellent tool to monitor progress of interventions and identify problematic sub-areas in need of special action, which would not have been identified by other survey types that report only on higher-level overall coverage values [29], [48], [49]. With the effective use of our data and the implementation of targeted interventions, PMV performance will hopefully improve in the future, probably gradually and especially on those aspects and in those areas where interventions have been most successful. For example, if during future monitoring it is found that PMVs in certain SDs perform adequately for key indicators, while others show inadequate performance, the latter might be able to learn from the experience of successful SDs. By empowering program managers to make data-driven decisions at decentralized levels, LQAS enables rapid responses targeted to the local context. LQAS will also be useful to compare pooled results of different geopolitical zones of the country, given that the population, culture, customs, and also certain aspects of the health care market differ between different states [4], [9].

We also recommend that future PMV assessments be integrated to complement HFAs, to monitor progress towards achievement of international and national targets and to evaluate the effectiveness of strategies. Having evaluators assess the PMV store nearest to the previously examined HF is a practical way of integrating HF and PMV assessments. This type of dual assessment is possible even if our assumption that ACT demand could be highest at these PMV stores might not be true. By the time our study was implemented, no interventions targeting PMVs had taken place in Jigawa. Our study was conducted as part of a baseline assessment among public and private health care providers prior to the implementation of interventions of the Malaria Booster Program. It also pre-dated the roll-out of other projects, such as the AMFm.

In general, a number of strategies have been suggested that target supply and demand side influences, and the prevailing market environment [3], [13], [44], [53], [54], [55]. A recent review [12] summarized the limited evidence available concluding that interventions had the potential to increase malaria-related practices of PMVs in Africa, but that a country’s legal and market environment had to be well analyzed and buy-in from all main stakeholders had to be gained. A combination of different approaches and strategies, which includes continued training and supervision, could have great chance for success [12].

### Conclusion

There is an urgent need to regularly monitor and improve the availability and quality of malaria treatment and advice provided by PMVs in Nigeria. The irrational use of antimalarials in the ACT era revealed in this study bears a high risk of economic loss, death and development of drug resistance. A combination of strategies underpinned by descriptive and operational research that further examine relevant factors and indicators is necessary for an effective and sustainable collaboration with PMVs [12], [44], [53]. LQAS has been shown to be a suitable method to monitor malaria-related indicators among PMVs and should be further applied across the country to effectively improve service delivery.
